# An Evaluation of the *in vivo* Safety of Nonporous Silica Nanoparticles: Ocular Topical Administration versus Oral Administration

**DOI:** 10.1038/s41598-017-08843-9

**Published:** 2017-08-15

**Authors:** Martha Kim, Joo-Hee Park, Hyejoong Jeong, Jinkee Hong, Woo Sung Choi, Byung-Han Lee, Choul Yong Park

**Affiliations:** 1 Department of Ophthalmology, Dongguk University, Ilsan Hospital, Goyang, South Korea; 2 School of Chemical Engineering and Material Science, Chung-Ang University, Seoul, South Korea; 3 Laboratory Animal Center, Osong Medical Innovation Foundation, Cheongju, South Korea

## Abstract

Nonporous silica nanoparticles (SiNPs) have potential as promising carriers for ophthalmic drugs. However, the *in vivo* safety of ocular topical SiNPs remains unclear. This study investigated the *in vivo* safety of oral and ocular topical applications of 100 nm-sized SiNPs in Sprague-Dawley rats. The rats were divided into the following four groups: low-dose oral administration (total 100 mg/kg of SiNPs mixed with food for one week), high-dose oral administration (total 1000 mg/kg of SiNPs mixed with food for one week), ocular topical administration (10 mg/ml concentration, one drop, applied to the right eyes four times a day for one month), or a negative control (no SiNP treatment). The rats were observed for 12 weeks to investigate any signs of general or ocular toxicity. During the observation period, no differences were observed in the body weights, food and water intakes, behaviors and abnormal symptoms of the four groups. No animal deaths occurred. After 12 weeks, hematologic, blood biochemical parameters and ophthalmic examinations revealed no abnormal findings in any of the animals. The lack of toxicity of the SiNPs was further verified in autopsy findings of brain, liver, lung, spleen, heart, kidneys, intestine, eyeballs, and ovaries or testes.

## Introduction

Recent advances in particle engineering technology have expanded the applications of nanoparticles (NPs), which can now serve as novel drug carriers. Nonporous silica NPs (SiNPs), which are commonly used as additives in cosmetics, printer toners, packaging, and imaging, represent promising drug carrier systems^1^. The stable chemical structure, large surface to volume ratios, ease of surface modification, and tolerable biodegradability of SiNPs greatly increase their attractiveness in biological applications as nanocarriers^1^.

As is well known, ocular drug penetration is difficult due to extrinsic and intrinsic ocular barriers, such as the tear film, mucus barrier, tight junction of the corneal epithelium, hydrophilicity of the corneal epithelium, and hydrophobicity of the corneal stroma^2^. Therefore, nano-based drug carrier systems, such as SiNPs, are a promising field of research for efficient ocular drug delivery^3, 4^. Several recent reports described successful intraocular drug or gene delivery via various NPs, further increasing expectations regarding their potential^5–11^.

Despite the potential of NPs, such as SiNPs, a major concern is their safety in medical applications^12, 13^. Research has shown that inhalation of some SiNPs can damage pulmonary cells via the induction of oxidative stress^14^. Disturbance of calcium homeostasis of neuronal cells after SiNP exposure has also been previously reported^15^. Concerns about the intestinal toxicity and genotoxicity of SiNPs have also been raised^16^. Nevertheless, recent research pointed to the safety of SiNPs. For example, Ryu *et al*.^17^ reported that SiNPs had no toxic effects, including no such effects on internal organs, after dermal topical application of SiNPs for 90 days. In another report, SiNPs elicited only minimal biological toxicity in intestinal cells compared to marked toxicity caused by zinc oxide NPs^18^. Previously, our group reported the *in vitro* safety of SiNPs on cultured human corneal epithelial cells and keratocytes^19, 20^. In our previous study, various sized SiNPs (50, 100, and 150 nm) did not result in any significant impairment of cellular viability after exposure for 24 h to concentrations up to 100 μg/ml. It is likely that the nanotoxicity of SiNPs are cell-type dependent^13^. Different types of cells are exposed to SiNPs, depending on the route of SiNP administration. Therefore, independent evaluations of the nanotoxicity of ocular topical administration of SiNPs are necessary to resolve whether SiNPs are safe for ophthalmic uses.

The current study investigated the *in vivo* effects of oral and ocular topical SiNPs in rats over a 12-week period. General toxicity-related effects (e.g., changes in body weight and food and water intake) of the SiNPs, as well as specific effects on hematologic parameters, biochemical parameters, and vital organs, including eyeballs, were studied.

## Results

### Clinical observations

The rats were divided into four groups (*n* = 10 each): control (Group 1), low-dose oral intake (Group 2), high-dose oral intake (Group 3), and topical administration (Group 4). Each group contained an equal number of males and females (Table 1).

**Table 1:** Groups, route of administration and dose levels of silica nanoparticles.

Group	Route	Dose	Number of animals
G1 (Control)			5 males and 5 females
G2	oral	100 mg/kg	5 males and 5 females
G3	oral	1000 mg/kg	5 males and 5 females
G4	topical	0.1 ml q.i.d for 1 month	5 males and 5 females

**Figure 1:**
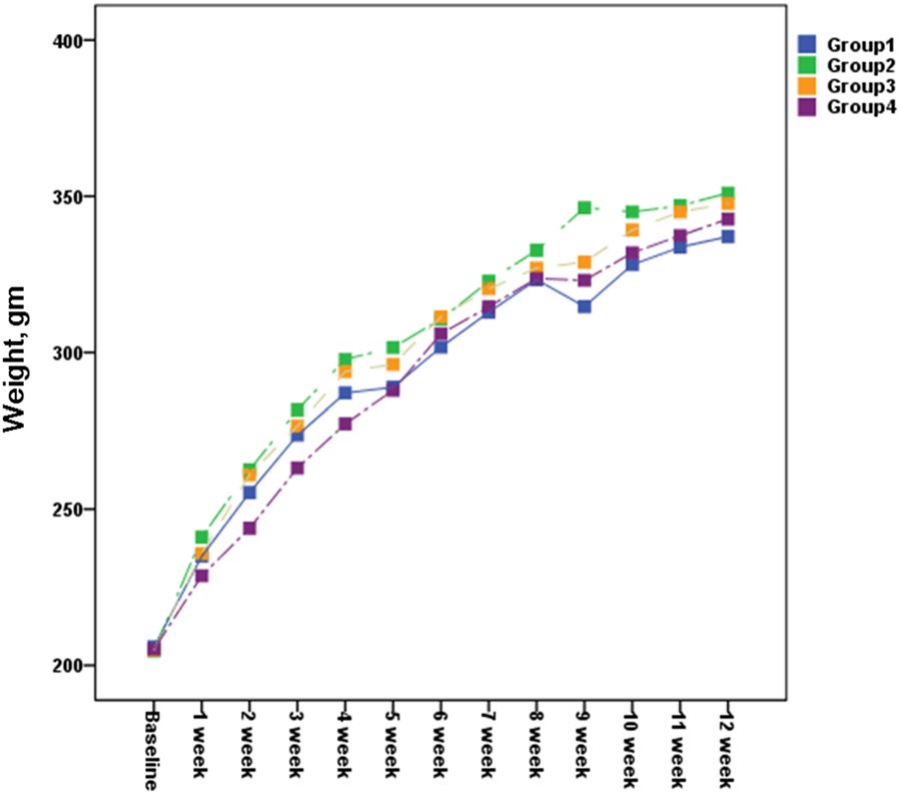
Body weight of the rats following the SiNP applications. There was no statistically significant difference in the body weights of the different groups (Group 1, control; Group 2, oral intake, 100 mg/kg; Group 3, oral intake, 1000 mg/kg; Group 4, topical application, 10 mg/ml) over the 12-week period.

Body weight, food and water intake, behaviors and abnormal symptoms (such as hyper/hypo-activity, abnormal breathing, abnormal locomotion, lethargy, paresis, paralysis, tremor, ruffled fur and eye discharge) were observed over a 12-week period after the administration of the SiNPs. No deaths occurred in any of the animal groups. As compared with the untreated controls, none of the groups showed body weight loss, differences in food and water intake, abnormal behaviors, or general abnormal symptoms (Table 2) (Fig. 1).

**Table 2:** Body weight (gram) changes of rats after SiNPs application up to 12 weeks.

		0 week	1 week	2 week	3 week	4 week	5 week	6 week	7 week	8 week	9 week	10 week	11 week	12 week
G1	Mean	205.9	234.9	255.3	273.7	287.1	288.9	301.7	312.9	323.3	314.7	328.1	333.7	337.1
	SD	38.5	45.5	56.0	63.5	67.9	70.9	71.4	75.1	87.4	78.8	83.9	86.7	89.0
	N	10	10	10	10	10	10	10	10	10	10	10	10	10
G2	Mean	204.6	241.0	262.6	281.7	297.8	301.6	310.7	322.8	332.7	346.3	345.0	347.0	351.0
	SD	39.2	56.6	65.0	75.1	81.5	83.8	85.7	90.4	91.8	103.9	96.4	99.2	103.7
	N	10	10	10	10	10	10	10	10	10	10	10	10	10
G3	Mean	204.9	235.8	261.0	276.6	293.9	296.2	311.4	320.4	327.0	328.9	339.2	345.0	347.7
	SD	39.1	50.3	59.2	70.4	76.1	79.3	78.9	86.9	89.5	92.1	97.7	99.4	103.8
	N	10	10	10	10	10	10	10	10	10	10	10	10	10
G4	Mean	205.2	228.7	243.9	263.2	277.3	287.9	306.0	314.6	327.7	323.1	331.9	337.4	342.7
	SD	38.9	46.3	50.1	54.4	63.1	65.9	65.5	75.4	77.5	81.0	83.3	87.9	89.3
	N	10	10	10	10	10	10	10	10	10	10	10	10	10

G: group, SD: standard deviation, N: number.

### Hematologic and biochemical examinations

The results of the hematologic and biochemical examinations 12 weeks after the application of the SiNPs are shown in Tables 1,2 and 3. There were no differences in the hematologic parameters of the oral intake groups (Groups 2 and 3). However, as compared to the control group (Group 1), there was a significant decrease in the mean platelet volume in Group 2. There were no differences in the hematologic parameters of the topical administration group (Group 4) compared to those of the oral intake groups. However, the mean platelet count (*P* < 0.01) and plateletcrit level (*P* < 0.05) of the topical administration group were significantly decreased compared to those of the control group (Table 3). There were no between-group differences in the differential count of white blood cells and reticulocytes (Table 4). Despite some significant differences between the groups, all the hematologic parameters were within normal ranges.

**Table 3:** Hematologic findings at 12 weeks (1).

		WBC × 10^3^/*μl*	RBC × 10^6^/*μl*	HGB g/dL	HCT %	MCV fL	MCH pg	MCHC g/dL	RDW %	HDW g/dL	PLT × 10^3^/*μl*	MPV fL	PDW %	PCT %
G1	Mean	7.52	7.99	14.4	47.3	59.3	18.1	30.5	12.64	2.27	995	8.68	48.64	0.86
	SD	3.75	0.66	0.9	4.1	2.5	0.7	0.9	0.66	0.20	110	0.42	1.91	0.09
	N	10	10	10	10	10	10	10	10	10	10	10	10	10
G2	Mean	5.69	7.97	14.3	46.5	58.5	18.1	30.9	12.38	2.17	943	8.21	48.44	0.78
	SD	1.99	0.54	0.4	2.3	2.1	0.9	0.9	0.46	0.15	100	0.55	1.46	0.12
	N	10	10	10	10	10	10	10	10	10	10	10	10	10
G3	Mean	5.61	8.01	14.6	47.1	58.9	18.2	30.9	12.61	2.23	907	8.50	49.74	0.77
	SD	2.18	0.62	0.6	3.0	2.5	0.8	0.8	0.41	0.13	88	0.50	1.15	0.11
	N	10	10	10	10	10	10	10	10	10	10	10	10	10
G4	Mean	5.87	7.92	14.5	46.3	58.6	18.3	31.2	12.58	2.24	837	8.64	49.77	0.73
	SD	2.49	0.48	0.8	2.3	3.0	0.9	0.8	0.44	0.15	105	0.49	2.16	0.12
	N	10	10	10	10	10	10	10	10	10	10	10	10	10

G: group, SD: standard deviation, N: number, WBC: white blood cells, RBC: red blood cells, HGB: hemoglobin, HCT: hematocrit, MCV: mean corpuscular volume, MCH: mean corpuscular hemoglobin, MCHC: mean cell hemoglobin concentration, RDW: red cell distribution width, HDW: hemoglobin distribution width, PLT: platelets, MPV: mean platelet volume, PDW: platelet distribution width, PCT: plateletcrit.

**Table 4:** Hematologic findings at 12 weeks (2).

		Neu %	Lym %	Mono %	Eos %	Baso %	Luc %	Neu × 10^3^/*μl*	Lym × 10^3^/*μl*	Mono × 10^3^/*μl*	Eos × 10^3^/*μl*	Baso × 10^3^/*μl*	Luc × 10^3^/*μl*	Reti %	Reti × 10^9^/L
G1	Mean	15.9	77.9	3.2	1.4	0.2	1.3	0.87	4.48	0.18	0.08	0.01	0.07	2.33	184.59
	SD	4.3	5.2	1.3	0.6	0.1	0.6	0.32	1.68	0.08	0.04	0.01	0.03	0.40	29.75
	N	10	10	10	10	10	10	10	10	10	10	10	10	10	10
G2	Mean	14.4	80.6	2.5	1.3	0.2	1.1	0.78	4.55	0.14	0.07	0.01	0.06	2.47	196.27
	SD	2.6	3.1	0.8	0.4	0.1	0.2	0.26	1.84	0.08	0.03	0.01	0.02	0.39	22.60
	N	10	10	10	10	10	10	10	10	10	10	10	10	10	10
G3	Mean	14.7	80.6	2.4	1.1	0.2	1.0	0.86	4.71	0.15	0.07	0.02	0.06	2.37	186.81
	SD	4.4	4.7	0.7	0.7	0.1	0.6	0.42	1.94	0.09	0.04	0.02	0.05	0.55	38.16
	N	10	10	10	10	10	10	10	10	10	10	10	10	10	10
G4	Mean	16.5	78.0	2.8	1.5	0.3	1.0	1.23	5.84	0.23	0.12	0.02	0.08	2.28	180.48
	SD	6.6	7.0	0.9	0.8	0.2	0.3	0.84	2.92	0.17	0.11	0.02	0.05	0.61	40.91
	N	10	10	10	10	10	10	10	10	10	10	10	10	10	10

G: group, SD: standard deviation, N: number, Neu: neutrophils, Lym: lymphocytes, Mono: monocytes, Eos: eosinophils, Baso: basophils, Luc: large unstained cells, Reti: reticulocytes.

Table 5 shows the biochemical findings 12 weeks after the treatments. In Group 2, gamma glutamyl transpeptidase (GGT), lactate dehydrogenase, creatinine kinase, and creatinine values were significantly decreased compared to those of the control group. In Group 3, GGT, total protein, albumin, and blood urea nitrogen values were significantly increased compared to those of the control group. As compared to those of the controls, the total protein, albumin, blood urea nitrogen, and low-density lipoprotein cholesterol values of Group 4 were also significantly increased. All the biochemical parameters were within normal ranges, despite statistically significant differences between the groups.

**Table 5:** Blood biochemical findings at 12 weeks.

		AST U/L	ALT U/L	ALP U/L	GGT U/L	LDH U/L	TBil mg/dL	DBil mg/dL	TPRO g/dL	ALB g/dL	BUN mg/dL	CREA mg/dL	TCHO mg/dL	TG mg/dL	HDLC mg/dL	LDLC mg/dL	GLU mg/dL	Na mmol/L	K mmol/L	Cl mmol/L	CK U/L
G1	Mean	129.09	58.37	316.28	5.69	409.87	0.14	0.13	5.83	3.08	15.62	0.37	83.26	73.76	69.77	18.46	262.88	142.18	4.25	102.61	116.50
	SD	38.94	10.56	89.86	1.15	146.47	0.03	0.02	0.28	0.11	1.40	0.04	12.23	38.25	10.03	4.65	86.98	2.05	1.05	1.60	25.98
	N	10.00	10.00	10.00	10.00	10.00	10.00	10.00	10.00	10.00	10.00	10.00	10.00	10.00	10.00	10.00	10.00	10.00	10.00	10.00	10.00
G2	Mean	102.65	52.10	290.40	4.83	279.19	0.13	0.11	6.11	3.14	16.71	0.34	84.88	59.86	64.50	19.00	234.59	144.43	3.75	103.84	86.60
	SD	23.31	7.08	67.16	0.45	97.29	0.02	0.02	0.43	0.22	2.29	0.03	13.91	21.55	5.87	3.94	19.70	0.94	0.68	1.50	31.05
	N	10.00	10.00	10.00	10.00	10.00	10.00	10.00	10.00	10.00	10.00	10.00	10.00	10.00	10.00	10.00	10.00	10.00	10.00	10.00	10.00
G3	Mean	119.01	50.33	315.23	4.81	403.88	0.13	0.12	6.47	3.25	17.63	0.36	89.73	61.25	72.49	20.99	253.78	143.72	4.02	102.99	121.63
	SD	45.88	9.64	48.59	0.44	190.07	0.02	0.01	0.40	0.16	2.00	0.02	16.17	24.82	14.42	4.58	42.30	1.30	0.96	1.03	38.77
	N	10.00	10.00	10.00	10.00	10.00	10.00	10.00	10.00	10.00	10.00	10.00	10.00	10.00	10.00	10.00	10.00	10.00	10.00	10.00	10.00
G4	Mean	116.94	54.03	318.82	5.18	349.81	0.14	0.12	6.40	3.23	19.44	0.37	90.34	63.95	71.07	23.02	240.60	143.59	3.97	102.88	105.65
	SD	30.02	7.93	67.77	0.57	85.56	0.02	0.01	0.36	0.18	3.60	0.03	12.47	31.29	11.15	4.76	51.44	1.10	0.77	1.18	18.29
	N	10.00	10.00	10.00	10.00	10.00	10.00	10.00	10.00	10.00	10.00	10.00	10.00	10.00	10.00	10.00	10.00	10.00	10.00	10.00	10.00

G: group, SD: standard deviation, N: number, AST: aspartate aminotransferase, ALT: alanine aminotransferase, ALP: alkaline phosphatase, GGT: gamma glutamyl transferase, LDH: lactate dehydrogenase, TBil: total bilirubin, DBil: direct bilirubin, TPRP: total protein, ALB: albumin, BUN: blood urea nitrogen, CREA: creatinine, TCHO: total cholesterol, TG: triglyceride, HDLC: high density lipoprotein, LDLC: low density lipoprotein, GLU: glucose, CK: creatinine kinase.

### Ophthalmic examinations

**Figure 2:**
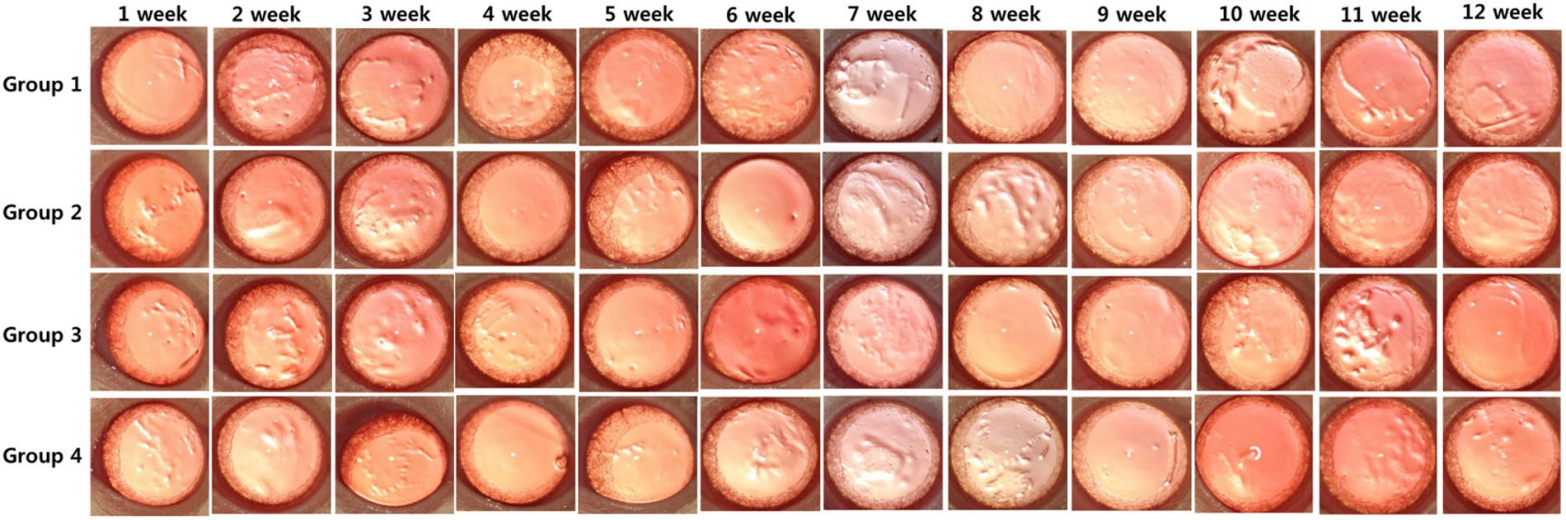
*In vivo* corneal effects of the SiNP applications. Digital photographs were obtained every week for 12 weeks. Corneal transparency and limbal vessels were normal in all the treated groups (Group 2, oral intake 100 mg/kg; Group 3, oral intake 1000 mg/kg; Group 4, topical application 10 mg/ml) as compared to those of the controls (Group 1).

**Figure 3:**
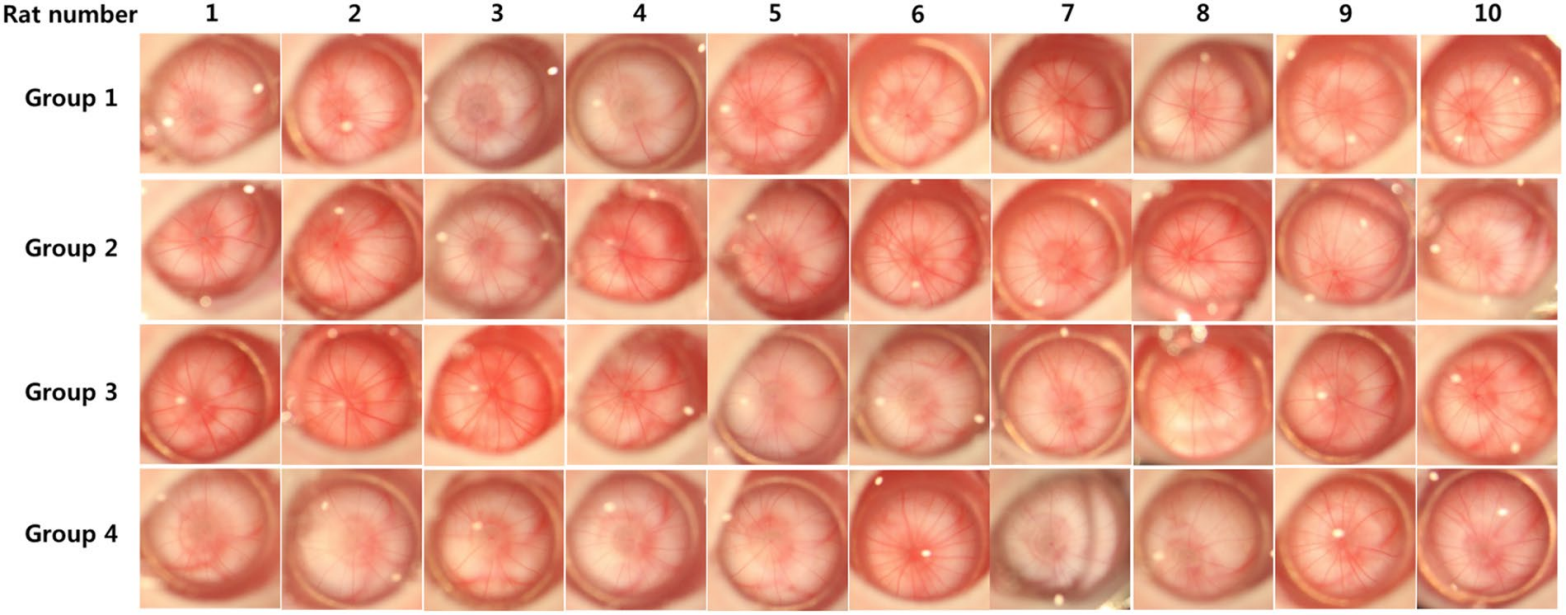
*In vivo* imaging of the optic nerve head following the SiNP applications. All the images were obtained 12 weeks after the treatments. There was no evidence of optic nerve damage or vascular changes in any of the treated groups (Group 2, oral intake 100 mg/kg; Group 3, oral intake 1000 mg/kg; Group 4, topical application 10 mg/ml) compared to the controls (Group 1).

In each group, the ocular surfaces were observed under surgical microscopic system closely each week. There was no ocular discharge, corneal opacity, or lid abnormalities in any of the groups. Digital photographs of the ocular surfaces showed no difference between the treated and control groups (Fig. 2). Corneal transparency was well maintained in all groups, with normal limbal vasculature. Fundus photographs taken monthly revealed no optic nerve or retinal abnormalities, such as hemorrhages and exudates, in either the treated or control groups (Fig. 3).

### Organ weight measurements and pathological examinations

**Figure 4:**
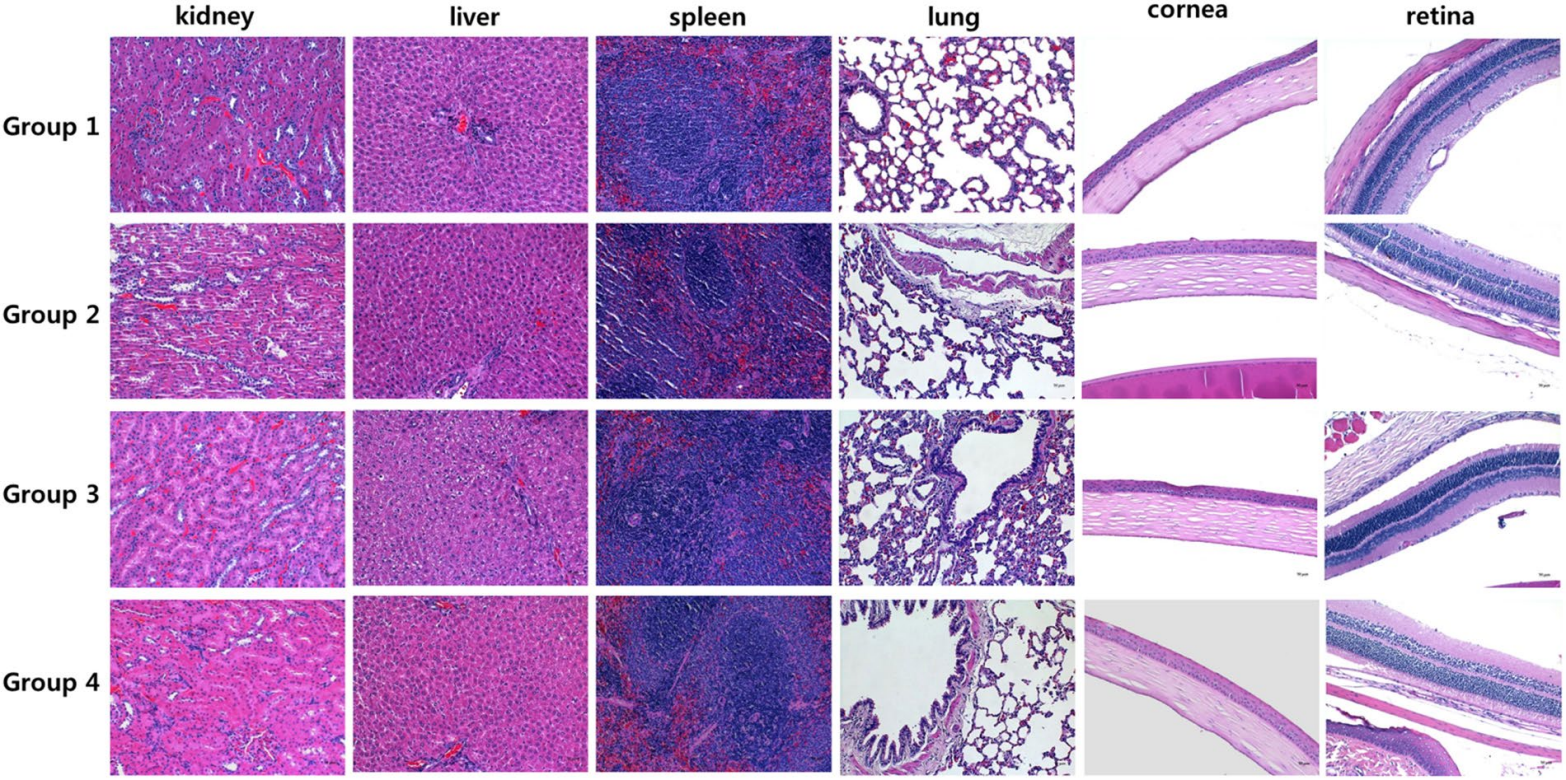
Histopathological findings of organs from the rats treated with SiNPs. Kidney, liver, spleen, lung, and eyeball samples were collected 12 weeks after the SiNP applications. Sections were stained with hematoxylin and eosin (×200): No treatment (Group 1, controls); low-dose oral intake (Group 2, 100 mg/kg); high-dose oral intake (Group 3, 1000 mg/kg); topical application (Group 4, 10 mg/ml).

The organ weights of all the treated groups were not significantly different in comparison to those of the control group. The pathological examinations revealed no toxicological effects of the SiNPs. Figure 4 presents biopsy findings of kidney, liver, lung, spleen, and eyeballs from the SiNP-treated groups. No abnormal changes were observed in Group 2 (SiNPs, 100 mg/kg), Group 3 (SiNPs, 1000 mg/kg), or Group 4 (10 mg/ml concentration, topical administration group), as compared to Group 1 (control).

## Discussion

This 12-week study investigated the *in vivo* effect of nonporous SiNPs after ocular topical administration, as well as oral intake. Based on general signs and symptoms and evaluations of target organs, including ocular tissues, small sized (100 nm) SiNPs induced no significant toxicity. To our knowledge, this is the first toxicity study to compare an ocular topically applied SiNP group with a control group and oral administration groups given different doses of SiNPs.

Daily human intake of silica nanoparticles from food is estimated to be 1.8 mg/kg according to a previous study^21^. When considering the initial body weight of the rats (approximately 200 g), our topical administration of SiNPs was equivalent to a daily application of 13.3 mg/kg onto the ocular surface. This is about 7.4 times higher than the estimated normal daily human oral exposure. In addition, we previously reported the an *in vitro* safety level of 100 μg/ml concentration of SiNPs onto cultured corneal epithelial cells^19^. The concentration tested in the current *in vivo* experiment (10 mg/ml) is 100 times higher than the verified safe concentration (100 μg/ml) for cell cultures. It is known that only 1% or less of topical medication reaches the target intraocular structure because of tear clearance and systemic absorption via conjunctival vessels^22^. This is why we selected the 10 mg/ml concentration of SiNP solution as the testing concentration. Our low and high doses of oral SiNPs are equivalent to daily exposures of 12.6 mg/kg and 126 mg/kg of SiNPs for one week. These are about 7 and 70 times higher than the estimated normal daily oral exposure in humans, respectively. Therefore, we consider our tested doses were high enough to reveal any toxicity of SiNPs that could be induced by ocular topical or oral applications as possible future drug delivery platforms.

Our results revealed no signs of acute or subchronic toxicity in the low-dose or high-dose oral intake SiNP groups. Furthermore, the high-dose (1000 mg/kg) treatment had no effects on body weights and food/water consumption. Lee *et al*.^23^ reported that kidneys, liver, lungs, and spleen were target organs of colloidal SiNPs (20 nm and 100 nm) administered via the oral route in rats. In the present study, the histopathological examinations revealed no target organ damage. In addition, the organ weights of the different groups were not significantly different. Although the hematologic and biochemical parameters of the groups varied slightly, these variations did not seem to have any particular significance. Together, these data suggested that oral treatment with nonporous SiNPs exerted no toxicity when administered at doses up to 1000 mg/kg for one week.

Several previous studies reported controversial results regarding the oral toxicity of SiNPs. Hassankhani *et al*.^24^ reported that oral administration of SiNPs with diameters of 10–15 nm resulted in significant changes in hematologic and biochemical parameters, such as albumin, cholesterol, triglyceride, total protein, urea, high-density lipoprotein, low-density lipoprotein, alkaline phosphatase, and aspartate aminotransferase activity in mice. They also demonstrated that SiNPs had toxic effects on the liver, kidneys, lungs, and testes. In contrast, Kim *et al*.^25^ reported that high-dose oral intake (2000 mg/kg) of 20- and 100-nm SiNPs for 90 days appeared to be relatively safe in rats. The inconsistency in the aforementioned findings on the oral toxicity of SiNPs could be explained by the results of previous studies, which found that the nanotoxicity of SiNPs was dependent on their size and dose, as well as the cell type^13, 26^. Our current result reconfirmed the safety of oral intake of 100 nm sized SiNPs.

The potential toxicity of SiNPs when used as drug carriers for ophthalmic drugs is a major challenge. To resolve this issue, toxicity data on topically administered SiNPs are needed. In the present study, topical application of SiNPs did not have toxic effects on the ocular surface, retina, or optic nerve. None of the animals showed any general toxicity-related signs, such as changes in body weight, food and water consumption, or behaviors. In addition, the topical SiNPs treatment did not cause target organ toxicity, as revealed by hematologic, biochemical, and histopathological examinations. These results indicated that SiNPs could be a safe candidate platform for topical drug delivery to ocular tissues.

Recently, Jo *et al*.^27^ reported that an intravitreal injection of SiNPs produced no toxic effects on retinal neuronal cells, retinal endothelial cells, and retinal tissues in mice. In addition, they demonstrated that the SiNPs effectively inhibited anomalous retinal angiogenesis in a mouse model of oxygen-induced retinopathy. Considering that neovascularization plays a pivotal role in various ophthalmic diseases^28–30^, these results suggested that SiNPs could be used not only as drug carriers but also as treatment modalities for eye diseases.

The present study contains several limitations. First, we tested only one size of SiNPs. Different sizes of SiNPs could have different toxicity-related effects. Secondly, testing only one concentration (10 mg/ml) of ocular topical SiNPs may not be enough to clearly resolve the safety issues. It is possible that unexpected toxicity could be found in much higher concentrations of SiNPs. However, as shown in Supplementary Figure 1, the tested concentration (10 mg/ml) of SiNPs made the solution turbid, and a more condensed concentration could create inadequate particle dispersion for a topical agent. In addition, we did not obtain bio-distribution and excretion data in the present study. Further studies are warranted to investigate these issues.

In conclusion, in this 12-week study, both ocular topical and oral administration of nonporous SiNPs for one month was safe. These findings suggest that both oral intake and topical administration of 100 nm-sized SiNPs are relatively safe for biomedical applications. The verification of the *in vivo* safety of SiNPs in the present study could be an initial step in the development of SiNPs-based topical drug delivery vehicles.

## Materials and Methods

### Preparation of the SiNPs

Nonporous SiNPs of 100 nm were prepared using the Stöber synthesis method. A detailed description of the manufacturing process of SiNPs has been reported previously^19, 20^. To synthesize SiNPs of 100 nm, 3 ml of ammonia (NH_4_OH, 28%, Junsei, Tokyo, Japan) and 50 ml of ethyl alcohol (EtOH, anhydrous, 99.5%, Daejung, Kyeonggi, Korea) were first mixed. Then, 1.5 ml of tetraethylorthosilicate (TEOS, Samchun, Kyeonggi, Korea) was quickly added while stirring the solution. Afterward, the solution was stirred for 12 h under ambient conditions (25 °C, 1 atm). The prepared SiNPs were washed three times with EtOH and centrifuged at 10,000 rpm for 15 min). The final SiNP precipitates were dispersed in distilled water.

### Animals

Sprague-Dawley rats (7 weeks old, 20 males and 20 females) were purchased from Koatech (Pyeongtek, Kyeonggi, Korea). The care and treatment of the animals complied with the ARVO Statement for the Use of Animals in Ophthalmic and Vision Research. The experimental protocol was approved by the Institutional Animal Care and Use Committee of Laboratory Animal Center, Osong Medical Innovation Foundation (reference number: KBIO-IACUC-2016–058).

### *In vivo* treatment with SiNPs

The rats in Group 1 received no treatment and served as the control. Groups 2 and 3 were treated with SiNPs (a total of 100 mg/kg and 1000 mg/kg, respectively) via the oral route. The SiNPs were mixed with food and administered for one week. In Group 4, the SiNPs were administered via a topical route (one drop of 10 mg/ml concentration diluted in phosphate buffered saline applied to right eyes four times a day for one month). All the rats were observed for 12 weeks.

### Clinical observations

Body weight and food and water intake were measured weekly. All signs of general toxicity were monitored daily.

### Hematology and biochemical tests

Blood samples were taken monthly from the carotid vein after anesthesia induced by isoflurane. The animals fasted for 12 h prior to the blood sampling. The hematologic measurements consisted of the blood cell count (white blood cells, red blood cells, and platelets), as well as hemoglobin and hematocrit values, differential count of white blood cells (neutrophils, lymphocytes, monocytes, eosinophils, and basophils), reticulocytes, mean corpuscular volume, mean corpuscular hemoglobin, mean cell hemoglobin concentration, red cell distribution width, and hemoglobin distribution width. The biochemical measurements consisted of analyses of the following: glucose, total protein, total cholesterol, triglyceride, low-density lipoprotein cholesterol, high-density lipoprotein cholesterol, alkaline phosphatase, alanine aminotransferase, aspartate aminotransferase, GGT, total bilirubin, direct bilirubin, blood urea nitrogen, creatinine, lactate dehydrogenase, creatinine kinase, and electrolytes (sodium, potassium, and chloride).

### Ophthalmic examination

In each group, the ocular surfaces were closely observed using surgical microscopic system (OPMI® Pico Surgical Microscope; Carl Zeiss, Oberkochen, Germany) each week. Any ocular discharge, opacity, or lid abnormalities were recorded. Digital pictures of the ocular surfaces were taken weekly using a surgical microscopic system. Fundus pictures were taken monthly using a surgical microscope and ocular contact lens system (G-4 gonio lens, Volk, Mentor, OH).

### Organ weight measurements and pathological examinations

At the end of the experiment, the animals were sacrificed, and their organs (brain, liver, lung, spleen, heart, kidneys [bilateral], intestine, eyeballs [bilateral], ovaries, or testes [bilateral]) were harvested. Close observation of any abnormal findings in all orifices, orbit, thorax, abdomen and cranial cavity was performed. Any signs of gross abnormalities were recorded. The weight of each harvested organ was measured as an absolute (organ itself) and relative (organ weight divided by body weight) value. The testes were fixed in Bouin’s solution, and the eyeballs were fixed in Davidson’s solution. The other organs were fixed with 10% neutral buffered formalin. The histologic examination was performed after hematoxylin and eosin staining.

### Statistical analysis

Data are presented as means ± standard deviation. Data on body weights, food and water intakes, hematologic parameters, biochemical parameters, and organ weights were evaluated by the Student’s *t*-test and an analysis of variance. The data were analyzed by SPSS (version 12.0; SPSS Inc., Chicago, IL, USA). Differences were considered statistically significant when *P*-values were less than 0.05.

## Electronic supplementary material

Supplementary Information
