# Supplementary material for: An Evaluation of the in vivo Safety of Nonporous Silica Nanoparticles: Ocular Topical Administration versus Oral Administration
Source: Sci Rep. 2017 Aug 15;7:8238. doi: 10.1038/s41598-017-08843-9 (PMC5557988; doi:10.1038/s41598-017-08843-9)

# An Evaluation of the *in vivo* Safety of Nonporous Silica Nanoparticles: Ocular Topical Administration versus Oral Administration

Martha Kim MD[1], Joo-Hee Park PhD[1], Hyejoong Jeong BS[2], Jinkee Hong PhD[2], Woo Sung Choi PhD[3], Byung-Han Lee PhD[3], Choul Yong Park MD, PhD[1]

**Supplementary information**

**Supplementary figure 1**. Silica nanoparticles (100nm sized SiNPs, 1mg/ml (A &B) and 10mg/ml (C&D) solution diluted with distilled water) before (A&C) and after sonication (B&D). SiNPs precipitate is clearly visible at the bottom of the tube before sonication (yellow arrows). Turbidity of SiNPs solution significantly increased after sonication (red arrows). DW: distilled water

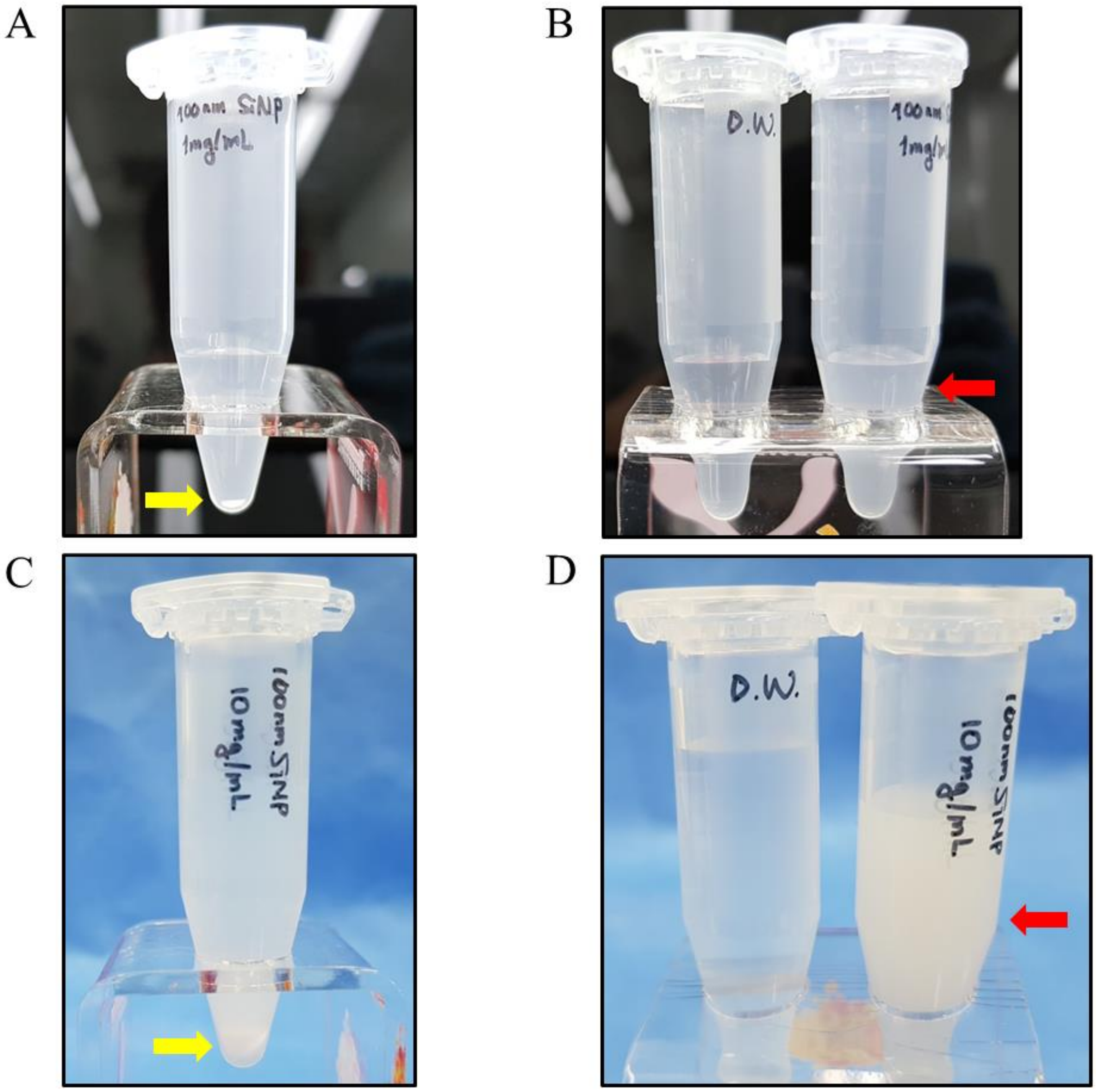

Supplement: Supplementary file 1 — Supplementary Information [file 41598_2017_8843_MOESM1_ESM.pdf]
